# Applying EEM- PARAFAC Analysis With Quantitative Real-Time PCR to Monitor Methanogenic Activity of High-Solid Anaerobic Digestion of Rice Straw

**DOI:** 10.3389/fmicb.2021.600126

**Published:** 2021-02-11

**Authors:** Yuying Deng, Weihua Li, Wenquan Ruan, Zhenxing Huang

**Affiliations:** ^1^Changzhou Vocational Institute of Engineering, Changzhou, China; ^2^School of Environmental and Civil Engineering, Jiangnan University, Wuxi, China; ^3^Anhui Provincial Key Laboratory of Environmental Pollution Control and Resource Reuse, Anhui Jianzhu University, Hefei, China

**Keywords:** anaerobic digestion, coenzyme F420, excitation emission matrix, methanogenic activity, rice straw

## Abstract

The methanogenic activity is an important indicator to assess the efficiency of high-solid anaerobic digestion. However, it is not yet elucidated clearly how to detect the parameter rapidly and reliably in the rice straw feeding reactor. Co-inoculated with ruminal digesta and anaerobic sludge, the digestion performance was studied at three different organic loading rates (OLRs). The excitation emission matrix–parallel factor analysis (EEM–PARAFAC) was used to detect dynamic changes in the characteristic of fluorescence components. Our results revealed that CH_4_ productivity reached 280.90 mL/g volatile solid (VS) with a 54.39% CH_4_ content under the OLR of 2.26 g/(L⋅d), which amount to 80.29% of its theoretical value. At the OLR of 2.47 g/(L⋅d), the average accumulated NH_4_^+^ concentration was 1082.63 mg/L, which resulted in the hydrogenotrophic *Methanobacteriales* decreasing from 1.70 × 10^9^ to 1.04 × 10^6^ copies/g in the solid residues, whereas the acetotrophic *Methanosarcinales* increased from 7.89 × 10^6^ to 9.44 × 10^6^ copies/g. The dynamics of the methanogenic community consequently influenced the bioconversion efficiency of rice straw, and CH_4_ productivity was reduced to 256.54 mL/g VS. The three fluorescent components, at the excitation/emission wavelength of 420 nm/470 nm, 340 nm/430 nm, and 280 nm/340 nm, were decomposed by PARAFAC model in the digestate. Fluorescence intensities of coenzyme F420 and NADH reflected the dynamic changes of CH_4_-producing activity and anaerobic digestion efficiency, respectively. The coenzyme F420, unique to hydrogenotrophic methanogens, was correlated with methane yield, suggesting they played a dominant role in the anaerobic reactor. This study demonstrates that the EEM–PARAFAC combined with Q-PCR can be used to characterize methanogenic activity variation during the high-solid anaerobic digestion of rice straw with 15% total solid (TS).

## Introduction

Rice straw, which consists mostly of cellulose, hemicellulose, and lignin, is among the organic components characteristic of plant materials. By analyzing all kinds of bio-transforming approaches for rice straw, Chandra et al. (2012) concluded that methanogenesis offers more thermodynamics advantages than do alcohol products via its anaerobic digestion of rice straw. Moreover, CH_4_-producing plants could lower straw incineration and acquire later use as a natural fertilizer. The traditional digestion process contains 6–8% total solid (TS) with 0.8 m^3^/(m^3^⋅d) volume biogas yield. Solid-state anaerobic digestion exceeding a 15% TS can alleviate the phenomena of crusting and stratification that occurs during a wet digestion; hence it is considered more suitable for straw degradation (Karthikeyan and Visvanathan, 2013). However, the commercial high-solid anaerobic process displays signs of “acid-intoxication” (Liotta et al., 2016) and ammonia limitation (Hu et al., 2014), among others, which affects the methanogens and subsequently their methane-producing activity. The dynamic changes of methanogenic activity are therefore an important indicator of the extent of anaerobic digestion. So, it would seem prudent to investigate the correlation between CH_4_ production and methanogenic activity. In our earlier study, the performance of anaerobic digestion had been investigated in a horizontal reactor; this work revealed that the CH_4_-producing efficiency is influenced by changes in the methanogens’ composition induced by detrimental metabolites (Deng et al., 2017b). Nevertheless, how to reliably detect and distinguish among methanogenic activity has yet to be extensively elucidated in real time during the entire digestion process.

Environmental samples contain soluble fluorescence peaks that appear within a certain range. According to Chen et al. (2003), an excitation wavelength (Ex) < 250 nm and emission wavelength (Em) > 350 nm (III area) are considered fulvic-like; conversely, those components ranging between Ex > 280 nm and Em > 380 nm (V area) are affiliated with humic-like qualities. Some microbial metabolites also have specific fluorescence peaks and they could be determined by the three-dimensional excitation emission matrix (EEM). The value at 280 nm/340 nm (Ex/Em) is the characteristic peak of the aromatic tryptophan (Chen et al., 2003; Pons et al., 2004; Li et al., 2008). The peak at 340 nm/460 nm or at 350 nm/450 nm is characterized by coenzyme NADH whereas coenzyme F420 functions as a unique electronic carrier in hydrogenotrophic methanogenesis, having a peak value at 420 nm/470 nm (Li et al., 2008, 2011b; Gao et al., 2015). Since the fluorescence intensities convey relative changes in the concentrations of components (Sheng and Yu, 2006), such characteristic peaks could be used to detect the fluorescence ones.

Parallel Factor Analysis (PARAFAC) can decompose the EEMs into their fluorescent components by reducing the interference present to provide more accurate intensity information. During the digestion of dehydrated sludge, the degradation order of tryptophan proteins, tyrosine proteins, and humic acids were reliably obtained according to their respective fluorescence intensities (Li et al., 2014). Earlier work showed that the concentrations of fluorescence proteins and NADH were moderately correlated with the degradation rate of sucrose and hydrogen-producing rate according to that EEM–PARAFAC analysis and a second-order calibration, which could be used to monitor both microbial status and anaerobic performance (Li et al., 2011a). By analyzing the fluorescence peaks, the fluorescence intensity of coenzyme F420 was found correlated with methane productivity in an inhibited reactor (Li et al., 2011b; Gao et al., 2015). Reducing NADH (i.e., the NADH/NAD^+^ ratio), which is involved in microbial redox reactions, could reflect the fermentation progress and digestion efficiency of participating anaerobic groups (Li et al., 2008; Zhang et al., 2019). Thus, a detailed assessment of fluorescent components during straw digestion could contribute to uncover the digestion efficiency in a reactor. Nevertheless, to the best of our knowledge, applying EEM–PARAFAC to the digestion of rice straw has not been reported in the literature.

Therefore, to fill this knowledge gap, this study aimed to investigate anaerobic digestion performance under the three organic load ratios (OLRs) with a 15 ± 2% TS. By means of EEM–PARAFAC and absolute quantitative PCR (Q-PCR), dynamic changes of the fluorescence components and microbial methanogens were analyzed. Correlations between measured fluorescence intensities of coenzyme F420 and CH_4_-producing activity were also investigated.

## Materials and Methods

### Inoculum and Substrate

Anaerobic sludge, containing 13.90% TS and 6.90% volatile solid (VS), was derived from an anaerobic reactor for treating kitchen waste (Jiangsu Clean Environment Technology Co., Ltd., China). The rumen digesta were collected from a raw cattle slaughterhouse (Wuxi, China) and consisted of 15.15% TS, 12.13% VS, 390.59 mg/L NH_4_^+^ concentrations and 200 mg/L butyrate. The anaerobic sludge and rumen digesta were co-inoculated with an equal VS ratio. Raw rice straw was collected from a paddy field (Xuzhou, China) and dried to a constant weight at 50°C in an air-circulating oven, and then stored in a vacuum bag after being crushed through a 20-mesh sieve. Its TS and VS were 96.91% and 86.12%, respectively.

### Semi-Continuous Horizontal Reactor

**FIGURE 1:**
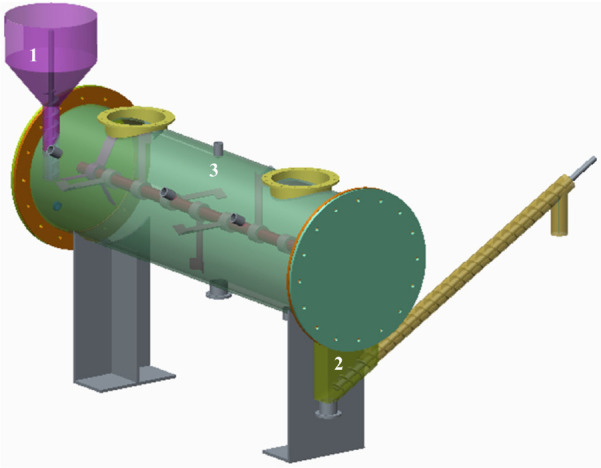
Schematic diagram of horizontal reactor. 1, feeding device; 2, discharging device; 3, biogas measuring device.

As depicted in Figure 1, the horizontal reactor used in this study featured semi-continuous charging and a discharging operating mode (Forster-Carneiro et al., 2008), with a 190-L working volume at 5 rpm/min. Three operating stage were set up with OLRs of rice straw being 1.89 g/(L⋅d), 2.26 g/(L⋅d), and 2.47 g/(L⋅d), whose corresponding weight of discharging digestate were 2.56 kg, 3.05 kg, and 3.41 kg at 2-day intervals, respectively. Each operating process lasted for 72 days, at 39°C, and the TS of digestate in the reactor fluctuated within the range of 15 ± 2%. The ensuing digestate was poured into a 100-mesh filter bag and then separated centrifugally in a dehydration device.

The liquid fractions were then added back into the reactor according to a 1:0.58 volume ratio with supplemented dH_2_O. Biogas productivity was measured at 2 days intervals and the methane content determined by the gasboard-3200 L infrared methane analyzer (Wuhan Sifang Photoelectric Technology Co., Ltd., China).

### Physicochemical Analysis

Both the TS and VS were measured according to American Public Health Association [APHA] (2005). The solid residues were dried at 105°C and then ground through a 100-mesh sieve. The C, N, H, and S contents of rice straw were determined in an elemental analyzer (vario MACRO, Elementar, Germany); the O element content was obtained by subtracting the proportion comprising the other four elements from the total value. The components of solid residue were determined every 9 days, by an ANKOM A2000i fiber analyzer (ANKOM Technology Co., United States). Neutral detergent fiber (NDF), acid detergent Fiber (ADF), and acid-insoluble lignin (ADL) were dried to a constant weight at 65°C. The liquid digestate was centrifuged at 5000 r/min for 15 min, and then diluted 10-fold with dH_2_O to determine its volatile fatty acids (VFAs) and NH_4_^+^ concentrations, in triplicate, every 4 days and 8 days, respectively. The VFAs were determined by using the GC-2010 Plus (Shimadzu, Kyoto, Japan) mixed with 0.83 g/L of 4-methylpentanoic acid and 3 mol/L of phosphoric acid. The temperature setting method followed that described in a previous study (Liu et al., 2008). The NH_4_^+^ concentrations were determined according to the Nessler reagent method (American Public Health Association [APHA], 2005).

### Fluorescence Component Analysis

To prevent interference by a filtration effect under high concentrations, the slurry was first diluted 100-fold and centrifuged at 12,000 r/min for 5 min. The supernatant was then filtered through a 0.45-μm membrane for further use. Fluorescence components were measured, every 9 days, with a three-dimensional fluorescence spectrometer (Hitachi F7000, Japan). Its parameters were set as follows: Ex spectra ranging from 250 to 450 nm, Em spectra ranging from 300 to 550 nm, and slit bandwidths of 5 nm with 2400 nm/min as the scanning speed. To avoid secondary Rayleigh scatter, a 290 nm cut-off filter was added to the side of emergent light.

In the three-dimensional fluorescence spectrum, the fluorescence peaks are superimposed each other and thus not accurately identified. To resolve this, PARAFAC was applied to obtain the characteristic fluorescence components and intensity information (Kowalczuk et al., 2010). To eliminate the Raman scatter effect, the Milli-Q water blank was subtracted from the tested samples, and then all the data were converted to an EEM dataset after setting the Rayleigh scatter peak to zero. By following the settings used by Li et al., 2011b, for fluorescence data analysis the DOMFluor toolbox was applied to MATLAB 7.0 software (MathWorks, Natick, MA, United States). Specifically, the number of fluorescence components was determined by using a split-half analysis and core consistency diagnostics. The fluorescence components of samples were identified by their excitation and emission wavelength loading scores, while the corresponding fluorescence intensities were obtained from the scoring matrix (Stedmon and Bro, 2008).

### Absolute Quantitative PCR (Q-PCR)

The samples were selected for DNA extraction every 24 days, by using PowerSoil^®^DNA isolation kit and following the manufacturer’s instructions. The primer sets according to Steinmetz et al. (2016) were used to amplify the DNA samples for their total methanogens, *Methanobacteriales*, and *Methanosarcinales*, respectively. The PCR procedure went as follows: initial denaturation at 95°C for 3 min, followed by 40 cycles of denaturing at 95°C for 30 s, annealing at 60°C for 30 s, extending at 72°C for 30 s; a final extension was made at 72°C for 10 min. After molecular cloning, the positive plasmid DNA was identified by sequencing of the samples (GENEWIZ, Inc., China). Plasmid DNA was serially diluted 10-fold, from 10^3^ to 10^9^ copies/μL; the 10-μL Q-PCR assay was set up with SYBR Premix Ex Taq^TM^ II (Takara Bio Inc., Dalian, China) that consisted of 0.2 μL of primer (10 μM) and 2 μL of plasmid DNA template. Each sample was repeated in triplicate, and the reaction procedure implemented according to a previous study (Deng et al., 2018). Consequently, the standard curve of plasmid copies against Ct values was established, from which the copies of target methanogens were calculated according to the Ct value in each sample. These results were expressed as copies per TS of solid residues.

### Statistical Analysis

The general formula C_*a*_H_*b*_O_*c*_N_*d*_S_*e*_ of rice straw was used to calculate the theoretical methane potential (TMP) (Pellera and Gidarakos, 2016). To detect significant differences among the three OLRs, one-way analysis of variance (ANOVA) was performed in SPSS 19.0 (SPSS Inc., Chicago, IL, United States), for which *p* = 0.05 served as the alpha threshold level.

## Results and Discussion

### Biogas and CH_4_ Production

**FIGURE 2:**
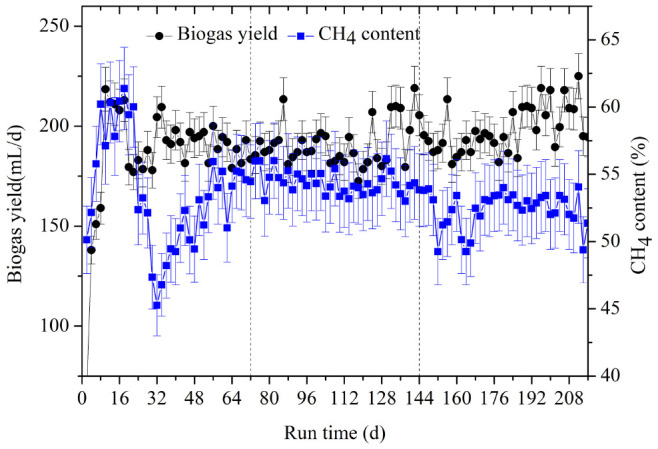
The dynamic changes of biogas yield and CH_4_ content at the three OLRs of 1.89 g/(L⋅d) (0–72 days), 2.26 g/(L⋅d) (73–144 days), and 2.47 g/(L⋅d) (145–216 days), respectively.

Biogas composition is an indicator that can be used to discern the decomposition of particular substrates. The changes in biogas yield and methane content are shown in Figure 2. From day 10 to 18 of the first OLR, there was a peak in biogas productivity ranging from 208 to 218.5 L/d. The methane content varied over a small range, from 57.13 to 61.38%, but then declined to its lowest value of 45.26% at day 32. From day 50 onward, the methane content was stable, at 51.02–55.95%. The methanogenic peak also appeared from day 10 to 18, followed by a decrease in CH_4_ productivity, which was only 271.85 mL/g VS at day 32. After 50 days, methane productivity had increased again, reaching the average of 321.88 mL/g VS for the whole stage (shown in Table 1). In the first OLR, the experimental co-inocula contained intrinsic proteins, soluble polysaccharides, and other easily degradable substances, which could be consumed first. According to the Buswell equation (Karthikeyan and Visvanathan, 2013), the general expression of carbohydrate is (C_6_H_10_O_5_)_*n*_ while that of protein is (C_5_H_7_O_2_)_*n*_, whose theoretical methane contents are 50% and 60%, respectively. Therefore, the higher CH_4_ content, ranging from 57.13 to 61.38%, in the first OLR mainly arose from the degradation of intrinsic proteins from rumen inoculum. As these substances are gradually consumed, the CH_4_ content and productivity began to decrease at day 32. After day 50, the methane-producing activity was enhanced, and the CH_4_ productivities gradually remained stable accompanied by the hydrolysis of rice straw.

**TABLE 1:** Changes of parameters in the horizontal reactor.

**Parameters**	**The first OLR**	**The second OLR**	**The third OLR**
CH_4_ productivities/(mL/g VS)	321.88 ± 53.21a	280.90 ± 16.61b	256.54 ± 17.54c
NH_4_^+^ concentrations/(mg/L)	877.74 ± 46.59c	950.80 ± 34.63b	1082.63 ± 57.11a
Intrinsic protein/(scores)	3844.91 ± 1637.36a	1753.84 ± 361.16b	1052.90 ± 194.16 b
Coenzyme F420/(scores)	2132.91 ± 645.64b	3042.43 ± 233.31a	2611.69 ± 138.44a
NADH/(scores)	2471.65 ± 590.71b	3171.00 ± 105.91a	3005.06 ± 135.80 a
The total methanogens/(copies/g TS)	1.38 (±0.31) × 10^11^a	3.62 (±2.95) × 10^10^b	3.47 (±3.52) × 10^8^b
*Methanobacteriales*/(copies/g TS)	1.20 (±1.46) × 10^10^a	1.70 (±2.33) × 10^9^a	1.04 (±0.49) × 10^6^a
*Methanosarcinales*/(copies/g TS)	4.24 (±1.70) × 10^6^a	7.89 (±10.10) × 10^6^a	9.44 (±7.75) × 10^6^a

*Mean (±SD) values with different superscript letters within the same row are significantly different at *p* < 0.05^a^.*

*^a^ Values are means ± SD (*n* = 36 for CH_4_ productivity, *n* = 9 for NH_4_^+^ concentration, *n* = 8 for fluorescence component, *n* = 3 for the copies of methanogens in each OLR).*

In the second OLR, the methane content varied from 53.01 to 56.16% and the average methane yield was stable at 280.90 mL/g VS with OLR rising from 1.89 to 2.26 g/(L⋅d). Theoretical CH_4_ productivity was calculated as 349.86 mL/g VS, according to the formula C_54_._88_H_97_._62_O_54_._43_NS_0_._04_ of rice straw, and it reached 80.29% of the theoretical value in this OLR. In the third OLR of 2.47 g/(L⋅d), the CH_4_ content decreased significantly to a minimum value of 49.25% from day 152 to day 170 (Figure 2), for which average CH_4_ content of the whole stage reached 52.36%. CH_4_ productivity first fell to 230.49 mL/g VS at day 166, and then increased slightly. The stage had a mean value of 256.54 mL/g VS.

The average CH_4_ yield reached 280.90 mL/g VS with the second OLR of 2.26 g/(L⋅d), which has a pronounced advantage when compared with the 92–280 mL/g VS during rice straw’s digestion after its physicochemical pretreatment reported in previous studies (Mussoline et al., 2013; Zhou et al., 2017). This remarkably high CH_4_-producing efficiency could be explained from several aspects. Firstly, full inoculations in the reactor were performed to increase the number of hydrolytic and methanogenic microbiota present, which would favor anaerobic digestion. Another also found that whole inoculation improved the digestion efficiency and methane production in the digestion of food waste material (Forster-Carneiro et al., 2008). Secondly, the recalcitrant structure of rice straw makes its hydrolysis notably difficult, which is the rate-limiting step in the whole anaerobic digestion process (Pellera and Gidarakos, 2016). The inoculum from rumen digesta contained myriad polysaccharide-degrading bacteria for improving the efficiency of hydrolysis (Yue et al., 2013). Further, co-inoculation of anaerobic sludge would have increased the fraction of methanogens and then enhanced the process of H_2_ transfer between the bacterial species (Demirel and Scherer, 2008). Finally, our previous results also support the finding that an addition of activated sludge could improve the efficiency of methanogenesis during the anaerobic digestion of rice straw (Deng et al., 2017a).

### Change of Solid Components

**FIGURE 3:**
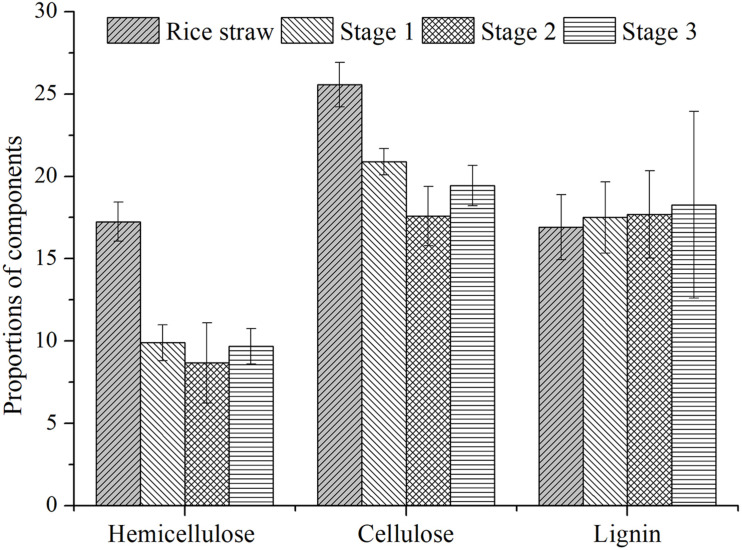
The dynamic changes of rice straw composition.

The altered composition of rice straw could reflect the hydrolytic efficiency of its solid residues. As Figure 3 shows, the hemicellulose fractions in residues of the three stages were 9.89%, 8.67%, and 9.69%, respectively; the corresponding proportions of cellulose were 20.88%, 17.58%, and 19.44%; likewise, for lignin composition they were 17.50%, 17.69%, and 18.27%, respectively. Compared with the components of raw materials, the degradation rates of hemicellulose were 42.62%, 49.71%, and 43.79%, respectively. The values of cellulose were 18.34%, 31.25%, and 23.99%, respectively. It was found that both hemicellulose and cellulose were degraded fastest in the second OLR, a result consistent with the latter’s CH_4_-producing rate. This also indicated the hydrolysis process was still the rate-limiting step in the anaerobic digestion of rice straw, for which a higher degradation rate promoted the CH_4_-producing efficiency. This CH_4_ was produced by the degradation of cellulose and hemicellulose rather than lignin composition (Chandra et al., 2012). Our study also supports this accepted conclusion, since no obvious degradation of lignin component was observed. In terms of rice straw structure, cellulose is enveloped by co-valent lignin and hemicellulose, which would prevent its degradation by hydrolytic bacteria (Mussoline et al., 2013). In our study, the degradation rate of hemicellulose exceeded that of cellulose; this not only exposed the micro-fibril structure of straw but also affected internal cellulose degradation (Vivian, 2011).

### Dynamics of VFAs and NH_4_^+^ Concentrations

**FIGURE 4:**
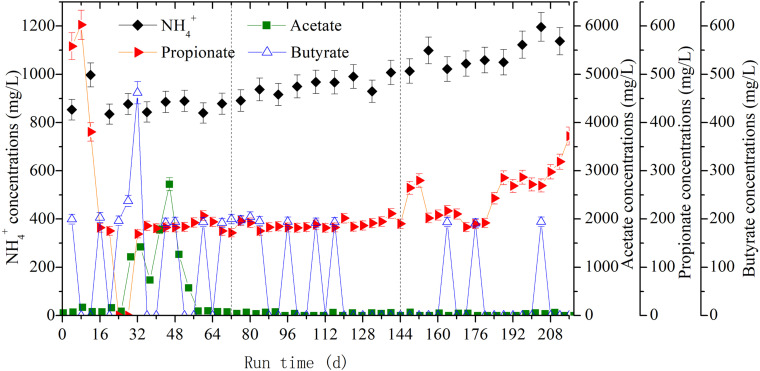
The dynamic changes of volatile fatty acids (VFAs) and NH_4_^+^ concentrations at the three OLRs of 1.89 g/(L⋅d) (0–72 days), 2.26 g/(L⋅d) (73–144 days), and 2.47 g/(L⋅d) (145–216 days), respectively.

The accumulation of individual VFA was used to investigate the metabolic balance between hydrolysis and methanogenesis. As seen in Figure 4, the acetate, propionate, and butyrate, generally referred to as VFAs, all accumulated during the anaerobic digestion of rice straw. Their respective average concentrations reached 597.82 mg/L, 218.01 mg/L, and 126.11 mg/L in the first OLR. With greater CH_4_ productivity in the second OLR, these average values decreased to 33.44 mg/L, 188.46 mg/L, and 65.43 mg/L, respectively. In the third OLR, the average concentration of accumulated propionate was 253.32 mg/L with an OLR of 2.47 g/(L⋅d). Especially from day 188 onward, its concentration increased, between 268.7 and 372 mg/L. As shown in Table 1, for the whole digestion process, the accumulated NH_4_^+^ concentrations had average values of 877.74 mg/L, 950.80 mg/L, and 1082.63 mg/L, respectively; noteworthy was the maximum of 1196.37 mg/L found at day 204.

By analyzing the VFAs-producing metabolic pathways under different OLR conditions, Lerm et al. (2012) found that incremental use of OLR would lead to accelerate butyrate oxidation. But when the threshold value exceeded metabolic capacity of methanogens, the H_2_ and reducing NADH could not be utilized in time and so they could only be released through the propionate-producing pathway. It follows that the concentrations of VFAs, especially propionate and butyrate, are important indicators for the inhibition of methanogenesis (Ahring et al., 1995). Accordingly, the accumulation of propionate in the third OLR indicated that methanogenic activity was inhibited, which is consistent with the corresponding CH_4_ productivity decreasing to 256.54 mL/g VS from 280.90 mL/g VS. Nitrogen, a vital nutrient for anaerobic microbiota (Mussoline et al., 2013), mainly came from the intracellular organic nitrogen released by rumen digesta in the present study. Moreover, it is generally recognized that NH_4_^+^ accumulates easily during the slurry recirculation process (Li et al., 2010; Mussoline et al., 2013), and this phenomenon also occurred in the anaerobic digestion of corn straw (Hu et al., 2014). We found an accumulated NH_4_^+^ concentration of 1082.63 mg/L in the third OLR of rice straw degradation. Although it did not attain the threshold value of 1500 mg/L for methanogenic inhibition (Karthikeyan and Visvanathan, 2013), it would nonetheless have significant toxicity effects upon hydrogenotrophic methanogens thereby restraining their methanogenic activity (Sprott et al., 1984; Demirel and Scherer, 2008).

### Shifts in the Fluorescence Components of the Digestate

**FIGURE 5:**
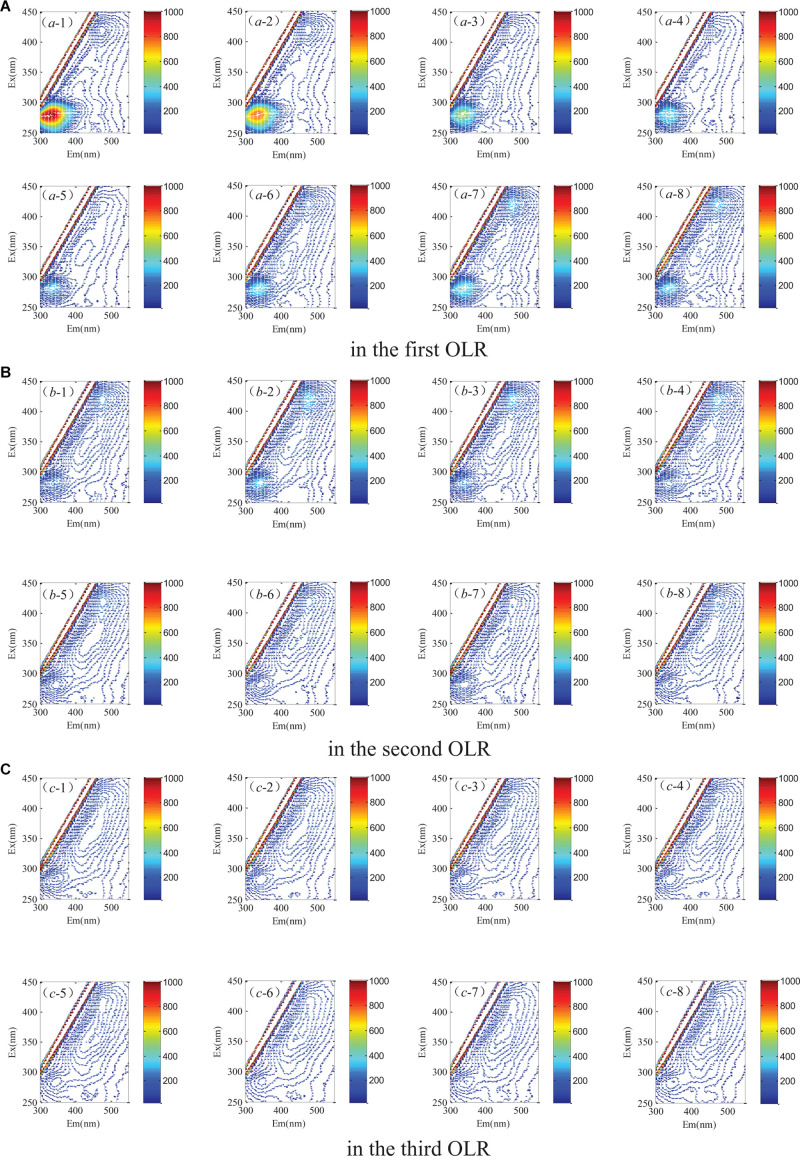
Spectral properties of the excitation emission matrix **(A)** in the first OLR, **(B)** in the second OLR, **(C)** in the third OLR of rice straw degradation.

**FIGURE 6:**
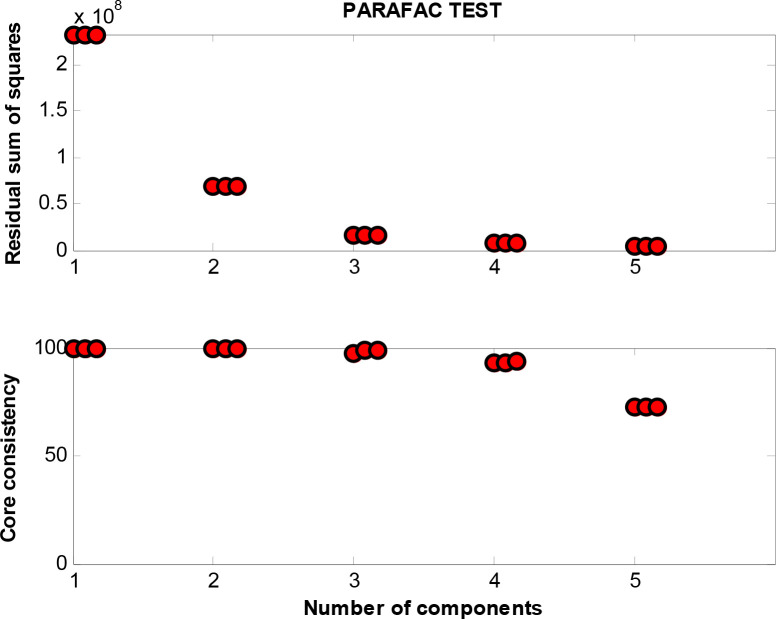
The residual sum of squares and core consistency of different number of components.

**FIGURE 7:**
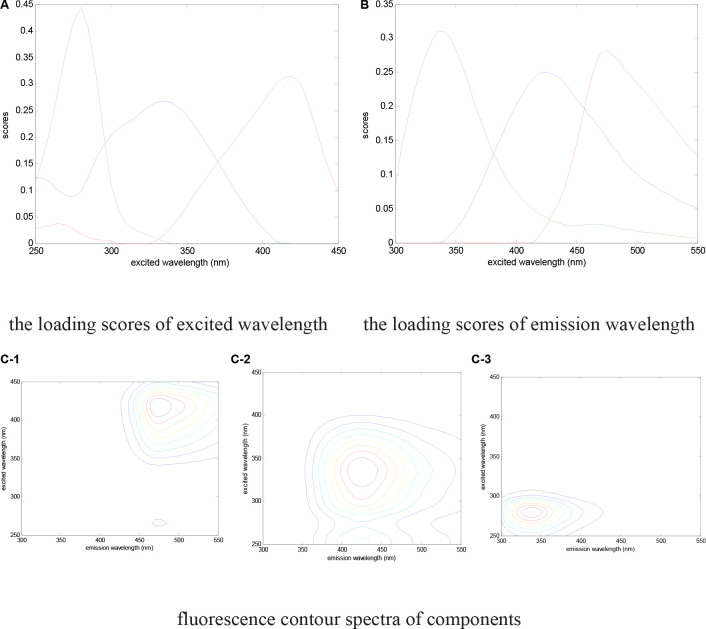
Fluorescence properties decomposed by the PARAFAC model. **(A)** The loading scores of the excited wavelength; **(B)** the loading scores of the emission wavelength; **(C)** fluorescence contour spectra of components.

Characteristic peaks could be used to determinate the fluorescence components. As Figure 5 shows, three peaks were detected by excitation emission matrix (EEM) spectra represented in the digestate. The determining process of model components is shown in Figure 6, where the fraction of the fluorescence components was determined by the analysis of residual sum of squares and core consistency. The former for the excitation spectrum decreased significantly as the group fractions increased to 3 from 2, indicating a better fitting model. In the ideal PARAFAC model, the core consistency would 100% (Bro, 1997). In present study, the value did reach 100% when the components were divided into three, whereas this value declined when divided into four (or five). Hence, the fluorescence peaks were ascertained as a three-component model. Subsequently, the fluorescence properties were decomposed (Figure 7) according to the loading scores of excitation and emission wavelengths in the applied PARAFAC model. The three fluorescence components were as follows: component 1 at 420 nm/470 nm (Ex/Em), component 2 at 340 nm/430 nm, and component 3 at 280 nm/340 nm; respectively, these components mainly contributed to coenzyme F420 (Li et al., 2008, 2011b), NADH with a slight offset (Li et al., 2008, 2011b; Gao et al., 2015), and aromatic conjugated structure in tryptophan-like (Pons et al., 2004; Li et al., 2014). The main source of component 3 in the present study was intrinsic proteins derived from the rumen inoculum.

**FIGURE 8:**
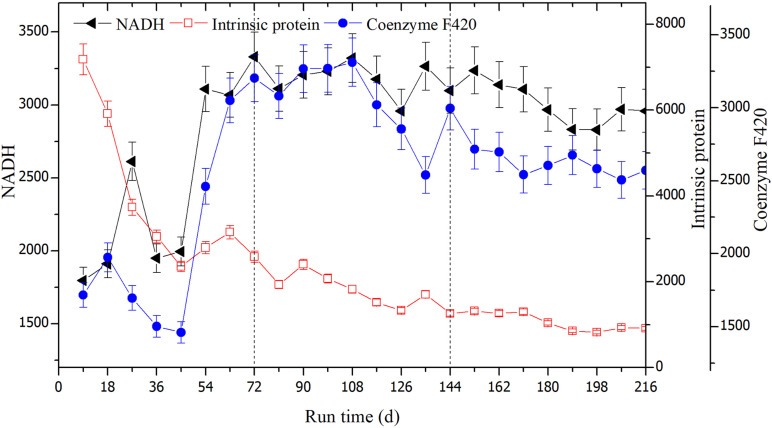
The changes in fluorescence intensities of the coenzyme F420, NADH, and intrinsic proteins at the three OLRs of 1.89 g/(L⋅d) (0–72 days), 2.26 g/(L⋅d) (73–144 days), and 2.47 g/(L⋅d) (145–216 days), respectively.

Fluorescence intensities could be used to detect the degradation of rice straw since the values were correlated with components. The changed fluorescence intensities are shown in Figure 8. The intensities of intrinsic proteins gradually weakened and their values tended to stabilize after day 45. The average scores of fluorescence intensities were 3844.91, 1753.84, and 1052.90 in the three successive OLRs, respectively. In the first OLR, fluorescence intensities of coenzymes F420 and NADH were the lowest, at 1459.90 and 1995.29, respectively. In the second OLR, these average values, respectively, increased to 3042.43 and 3171.00 (shown in Table 1). In the last OLR of rice straw degradation, the fluorescence intensity of coenzyme F420 initially decreased, but then recovered; its lowest value was 2542.01 at day 171. After day 189, the values of coenzyme F420 declined again, whereas the ones of NADH displayed the opposite trend.

The characteristic peak of intrinsic proteins is generally correlated with the microbial metabolites or decaying cells (Pons et al., 2004). In the first OLR, intrinsic proteins-like were probably decomposed first, leading to the emergence of a CH_4_-producing peak; both coenzyme F420 and NADH also showed their first peaks, corroborating this interpretation. After 45 days, the fluorescence intensities of intrinsic proteins tended to be stable, indicating they had been completely consumed. Meanwhile, NADH and coenzymes F420 reached their lowest values. With the hydrolysis of rice straw, the fluorescence intensities of the two components began to rise. In the second OLR, the two values increased sharply along with CH_4_-producing activity and higher digestion efficiency. Since coenzyme F420 is a unique electron transfer carrier in hydrogenotrophic methanogenesis, it is often used to characterize methanogenic activity (Li et al., 2011b; Gao et al., 2015). In an earlier study, there was a linear correlation between the intensities of coenzyme F420 and number of methanogens in the anaerobic digestion of hot, pretreated kitchen waste (Wang et al., 2005). Using the EEM–PARAFAC and biochemical indexes, Li et al. (2011b) confirmed the fluorescence intensities of coenzyme F420 are associated with methane productivities in an anaerobic reactor. By analyzing the its fluorescence peaks, it was also shown that coenzyme F420 reflected the changed CH_4_ yields in an NH_4_^+^ inhibited reactor (Gao et al., 2015).

In the last OLR, the fluorescence intensities of coenzyme F420 began to decline due to the NH_4_^+^ accumulation, indicating the hydrogenotrophic methanogenesis was restrained. This trend was basically consistent with our results for the changes in CH_4_-producing yield. After day 189, the reducing NADH produced during the straw degradation process could not be utilized because methanogens were inhibited, resulting in its trend of increasing. Similar results were also reported for the inhibition of methanogenesis, these attributed to a high loading rate which led to a rapid pH decline, driving accumulation of reducing NADH and the decline of coenzyme F420 (Li et al., 2011b). In a recent study on the digestion of food waste and excess sludge, the reduced and oxidized forms of NADH were deemed an effective indicator of VFAs’ accumulation (Zhang et al., 2019). In a sequencing batch reactor, the intensities of intracellular NADH underwent similar shifts with oxygen uptake rate, suggesting this component was correlated with the specific substrate degradation rate, and microbial activity of sludge (Li et al., 2008). Reducing NADH, as the electron acceptor, could accumulate when the involved anaerobic consortia do not proliferate in the anaerobic hydrogen-producing reactor (Li et al., 2011a). Those findings are in line with trends we found in the last OLR of rice straw degradation.

### Changes in the Copies of Targeted Methanogens

Absolute Q-PCR was performed for the total methanogens, *Methanobacteriales* and *Methanosarcinales*, respectively. As shown in our earlier study, the actual fragment sequences had 100% similarity with identified methanogenic genera deposited in the GenBank (Deng et al., 2018). Their corresponding *R*^2^ values were 0.9986, 0.9958, and 0.9873, which indicated that the standard regression equation could be useful for making subsequent calculations. According to Table 1, the copies of the total methanogens in solid residues decreased gradually, yet there was little discrepancy evident between the second and third OLRs. The copies of *Methanobacteriales* decreased gradually in the three OLRs, which were 1.20 × 10^10^ copies/g TS, 1.70 × 10^9^copies/g TS, and 1.04 × 10^6^ copies/g TS, respectively. The copies of *Methanosarcinales* increased slightly, and the number in the third OLR was higher than that of *Methanobacteriales*.

The changes of methanogenic community were beneficial to illustrate how the OLRs influenced the methanogenic pathways. The *Methanobacteriales* members, including *Methanobacterium*, *Methanobrevibacter* and *Methanosphaera*, among others, are affiliated with hydrogenotrophic methanogens. The aceticlastic and methylotrophic *Methanosarcinales* mainly consist of *Methanosarcina* (Demirel and Scherer, 2008). Through shifts in the relative abundances of these two different types of methanogens, the influence of operating parameters on methanogenic pathway could be analyzed (Steinmetz et al., 2016). In the first two OLRs, it was showed the main methanogens were *Methanobacteriales*, which increased methanogenic activity by accelerating the interspecific H_2_ transfer, generating the increased fluorescence intensities of coenzyme F420. In a biogas plant loaded with rice straw, CH_4_ productivity improved with an OLR below 2 g VS/(L⋅d) or 2.18 g TS/(L⋅d). Similarly, it was found that hydrogenotrophic methanogens, such as *Methanomassiliicoccus*, *Methanospirillum*, and *Methanobacterium*, dominated the whole methanogenesis process (Zhou et al., 2017). In a previous study of co-inoculated anaerobic digestion of rice straw, the hydrogenotrophic *Methanobrevibacter* were able to form functional consortia with rumen hydrolytic bacteria, which enhanced the prospects of hydrolysis and methanogenesis occurring (Deng et al., 2017a). In an anaerobic reactor, the associated populations of *Methanobacteriales* formed functional groups with propionate and butyrate oxidizers, leading to a higher syntrophic efficiency (Deng et al., 2018).

It is known that methanogens are susceptible to environmental conditions. *Methanobacteriales*, having a rod-like structure and longer retention time, are easily inhibited by excessively high NH_4_^+^ concentrations. The free ammonia produced by ammonia nitrogen diffuses into the cell membrane and impairs the K^+^-ATPase activity (Sprott et al., 1984), eventually causing toxicity to those methanogens. By contrast, *Methanosarcinales* has strong environmental adaptability, often forming a polymer structure with irregular cell clumps and a short retention time (Demirel and Scherer, 2008). When the NH_4_^+^ concentration reached 1082.63 mg/L in the third OLR of this study, the copies of *Methanobacteriales* decreased markedly and the fluorescence intensities of coenzyme F420 also declined accordingly. Consequently, the CH_4_ productivity decreased to 256.54 mL/g VS. So, our study also supports the generally held view that hydrogenotrophic populations are crucial for sustaining anaerobic digestion (Lerm et al., 2012). By contrast, *Methanosarcinales* was only marginally inhibited, in that its copies increased to 9.44 × 10^6^ copies/g TS, thus suggesting acetotrophic methanogenesis became the dominant bioconversion pathway in the final OLR. During the digestions of pig manure, maize straw, and mixture feedstock, significant shifts in the methanogen community also occurred, with the OLR increasing to 4 g/(L⋅d), but the hydrogenotrophic and aceticlastic methanogens were only found in approximately equal contents for the digestion of maize straw (Kong et al., 2018). Its corollary is that the reduced hydrogen produced during the hydrolysis process may not be used by methanogens in a timely manner, causing propionate to accumulate in the reactor.

## Conclusion

Semi-continuous experiment was designed to investigate anaerobic digestion performance with three different OLRs [1.89 g/(L⋅d), 2.26 g/(L⋅d), and 2.47 g/(L⋅d)]. The CH_4_ was not produced by the hydrolysis of rice straw until 50 days. The average methane yield of rice straw reached to 280.90 mL/g VS with around 54.39% CH_4_ content at the OLR of 2.26 g/(L⋅d), which is up to 80.29% of the theoretical yield. Hemicellulose and cellulose were degraded fastest in this OLR, reaching 49.71% and 31.25%. Three fluorescent components were decomposed by using a split-half analysis and core consistency diagnostics on PARAFAC model. During the whole digestion, fluorescence intensities of coenzyme F420 (420 nm/470 nm, Ex/Em) and NADH (340 nm/430 nm, Ex/Em) reflected the dynamic changes of methanogenic activities and anaerobic digestion efficiency, respectively. After 45 days in the first OLR, the fluorescence intensities of intrinsic proteins (280 nm/340 nm, Ex/Em) tended to be stable. The copies of the total methanogens in solid residues decreased slightly during the three OLRs. The copies of *Methanobacteriales* decreased gradually, being 1.20 × 10^10^ copies/g TS, 1.70 × 10^9^ copies/g TS, and 1.04 × 10^6^ copies/g TS, respectively. By contrast, the copies of *Methanosarcinales* increased slightly. At the OLR of 2.47 g/(L⋅d), the NH_4_^+^ concentration accumulated to average 1082.63 mg/L, which resulted in hydrogenotrophic methanogenesis being restrained. Consequently, the CH_4_ productivity decreased to 256.54 mL/g VS. The average concentration of accumulated propionate was 253.32 mg/L accordingly. The hydrogenotrophic methanogenesis played a dominant role during the anaerobic digestion of rice straw. And fluorescence intensities of coenzyme F420 are therefore an important indicator of the extent of anaerobic digestion. Thus EEM-PARAFAC analysis with Q-PCR can be used to detect CH_4_-producing activity reliably during anaerobic digestion of rice straw.

## Data Availability Statement

The original contributions presented in the study are included in the article/supplementary material, further inquiries can be directed to the corresponding authors.

## Author Contributions

All authors listed have made a substantial, direct and intellectual contribution to the work, and approved it for publication.

## Conflict of Interest

The authors declare that the research was conducted in the absence of any commercial or financial relationships that could be construed as a potential conflict of interest.
